# Editorial Note: Does high-speed rail stimulate university technology transfer? evidence from China

**DOI:** 10.1371/journal.pone.0336074

**Published:** 2025-11-06

**Authors:** The PLOS One Editors

This article [1] was identified as one of a series of submissions for which we have concerns that the peer review did not meet the expected standards for *PLOS One*. We regret that the issues were not identified and addressed prior to the article’s publication.

This note is not intended to question the quality of the article or contributions of the authors to the review process.

Additionally, the following references were cited in [1] in response to reviewer requests, and the Editors are concerned about their relevance to this article: 1, 10, 11, 12, 13, 55, 57, 58, 59.

## References

[1] Wu X, Luo H, Wu Y. Does high-speed rail stimulate university technology transfer? evidence from China. PLoS One. 2023;18(5):e0285431. doi: 10.1371/journal.pone.0285431 37205677 PMC10198495

